# Data on the interaction between thermal comfort and building control research

**DOI:** 10.1016/j.dib.2018.01.033

**Published:** 2018-02-02

**Authors:** June Young Park, Zoltan Nagy

**Affiliations:** Intelligent Environments Laboratory, Department of Civil, Architectural and Environmental Engineering, The University of Texas at Austin, Austin, TX, USA

## Abstract

This dataset contains bibliography information regarding thermal comfort and building control research. In addition, the instruction of a data-driven literature survey method guides readers to reproduce their own literature survey on related bibliography datasets. Based on specific search terms, all relevant bibliographic datasets are downloaded. We explain the keyword co-occurrences of historical developments and recent trends, and the citation network which represents the interaction between thermal comfort and building control research. Results and discussions are described in the research article entitled “Comprehensive analysis of the relationship between thermal comfort and building control research – A data-driven literature review” (Park and Nagy, 2018).

**Specifications Table**

**Table:** 

Subject area	*Engineering*
More specific subject area	*Thermal comfort and building control*
Type of data	*Text file and table*
How data was acquired	1. *Downloaded from Web of Science bibliographic database* 2. *Processed using VOSviewer software*
Data format	*Raw and analyzed*
Experimental factors	*N/A*
Experimental features	*N/A*
Data source location	*N/A*
Data accessibility	*Data is with this article*

**Value of the data**

• The bibliographic information for thermal comfort and building control research articles (5536 papers)
• keywords co-occurrences for describing the historical development and recent trend for both thermal comfort and building control research
• Citation network for investigating the interaction between thermal comfort and building control research
• Instruction to reproduce our data-driven literature review method

## 1 Data

The dataset contains 3 folders: 1) The first folder is all the bibliographic information for thermal comfort and building control research. The total number is 5536 articles, and the publication range is from 1970 to 2016. Table 1 summarizes general information about the publications for the two different search periods. The bibliographic information is summarized by multiple text files. 2) The co-occurrences among keywords are described. Firstly, the keywords are extracted from the title and abstract text and they are further filtered by pre-defined thesaurus words. Subsequently, the keywords are clustered based on research topics. Finally, the co-occurrences among keywords are normalized as distances among them. The files contain each keyword and its coordinate for the two periods. For the visualization of this two, the figures can be found in the original research paper [1]. Table 2, Table 3 explain keyword analysis for historical developments and recent trends, respectively. 3) The papers essentially are classified by their research theme (i.e., thermal comfort, building control, both), and their citation relationship is tabulated by matrix form in the data. Table 4 describes the citation relationship among the three themes (i.e., thermal comfort, building control, intersection). Note that only 3572 papers build the citation relationship. This citation relationship is also visualized in the original research paper [1].

**Table 1:** Raw data summary for the publications.

Topics	Thermal comfort	Building control	Both topics
Nr of papers	3707	1951	122
Nr of publication sources	1978 (*15 top sources account for 34% of the whole publications)		
Available bibliographic information	Title, abstract, authors, publication sources, citations

**Table 2:** Keyword analysis for the historical developments (-2010).

Nr of topics	4 (thermal comfort, material, building control, ventilation)
Nr of keywords	80
Top five occurred keywords (times)	thermal comfort (1746), building control (705), energy consumption (609), occupant (606), environment (602)
Nr of thesaurus words	361

**Table 3:** Keyword analysis for the recent trends (2011–2016).

Nr of topics	6 (thermal comfort, material, building control, ventilation, urban, indoor environmental quality)
Nr of keywords	155
Top five occurred keywords	thermal comfort (3403), energy demand (1692), occupant (1414), energy (1398), data (1250)
Nr of thesaurus words	691

**Table 4:** Citation relationship among research themes.

	Cited by themes below		
	Thermal comfort	Building control	Intersection
Thermal comfort	2407	305	87
Building control	195	130	33
Intersection	92	21	60

## 2 Methods

### 2.1 Data collection

For the publication collection, we selected Thomson Reuters' Web of Science bibliographic database [2]. We used the following logical combinations of search terms to collect relevant publications: For thermal comfort research related to buildings, we use the search term

(thermal comfort) AND (building*)

On the other hand, the search term for building control research related to energy efficiency was

**Table:** 

(building* automation*)	OR (building* energy management*)
	OR (building* control*)
	OR (HVAC control*)

owing to the fact that building control research can be found under several alternative terms. Using these search terms, we downloaded the publication information, i.e., title, abstract, author, citation, publication year, as a tab-delimited text file, suitable for further processing. Essentially, we split the dataset into two parts by publication dates. The first contains all the publications until 2010 and allows us to study the historical developments. The second part is for the publications from 2011–2016 in order to identify recent trends.

### 2.2 Keyword analysis

For the selection of keywords in a scientific landscape, all the words were extracted from the title and abstract of the publication collections and they were filtered for a minimum of 30 occurrences. With filtered words, the most relevant keywords were extracted through a VOSviewer built-in text mining function [3], [4]. Subsequently, we eliminated unrelated words (i.e., regional words, organization names, generic terms) and merged repetitive words (i.e., singular and plural forms, and abbreviation and full name) by applying the pre-defined thesaurus files. With the list of keywords, the VOSviewer generated the co-occurrence table and clustered the keywords based on the co-occurrences. Two words are defined as co-occurred if they appear in the same document. In addition, the cluster names were manually labeled based on the observed keywords.

Ultimately, the scientific landscape of thermal comfort and building control research is generated. In this figure, the size and color of the circle represents the frequency of occurrence and cluster type of the individual keyword, respectively. Lastly, the distance between the keywords is representative of their relative co-occurrence, e.g., two keywords that are close to each other co-occur more frequently, whereas a large distance between two keywords indicates that they do not co-occur.

### 2.3 Citation analysis

To identify the interaction between thermal comfort and building control research, we investigate citations of the whole publications. Analyzing citation information specifies quantitative interactions between the two (i.e., number of publications cited by others).
